# Comprehensive Gene Mutation Profiling of Circulating Tumor DNA in Ovarian Cancer: Its Pathological and Prognostic Impact

**DOI:** 10.3390/cancers12113382

**Published:** 2020-11-16

**Authors:** Tomoko Noguchi, Naoyuki Iwahashi, Kazuko Sakai, Kaho Matsuda, Hitomi Matsukawa, Saori Toujima, Kazuto Nishio, Kazuhiko Ino

**Affiliations:** 1 Department of Obstetrics and Gynecology, Wakayama Medical University, 811-1 Kimiidera, Wakayama 641-0012, Japan; naoyuki@wakayama-med.ac.jp (N.I.); ka-matsu@wakayama-med.ac.jp (K.M.); hitomi_m@wakayama-med.ac.jp (H.M.); toujima@wakayama-med.ac.jp (S.T.); kazuino@wakayama-med.ac.jp (K.I.); 2 Department of Genome Biology, Kindai University Faculty of Medicine, 377-2 Ohno-higashi, Osaka-Sayama, Osaka 589-8511, Japan; kasakai@med.kindai.ac.jp (K.S.); knishio@med.kindai.ac.jp (K.N.)

**Keywords:** circulating tumor DNA (ctDNA), gene mutation profile, liquid biopsy, ovarian cancer, prognosis

## Abstract

**Simple Summary:**

Recent advances in cancer genomic medicine enabled gene-profiling of individual tumors using tumor tissue DNA. However, surgical tumor biopsy is invasive and sometimes difficult to perform in advanced/recurrent cancers. Liquid biopsy using circulating tumor DNA (ctDNA), which can analyze in real time and repeatedly, has attracted attention as a non-invasive technique, although it has been rarely used in ovarian cancer. The aim of the present study was to demonstrate the comprehensive gene mutation profiles of ctDNA in ovarian cancer patients with different histological subtypes and its association with clinicopathological and prognostic outcomes. Of 51 patients, 48 showed one or more non-synonymous somatic mutations, including *TP53*, *APC*, *KRAS*, *EGFR*, *MET*, and *PIK3CA*. Patients with higher ctDNA concentration or with any pathogenic mutations showed worse progression-free survival (PFS). These results suggest that ctDNA-based gene profiling may serve as a prognostic indicator and might help in establishing personalized therapeutic strategies for ovarian cancer.

**Abstract:**

Liquid biopsies from circulating tumor DNA (ctDNA) have been employed recently as a non-invasive diagnostic tool for detecting cancer-specific gene mutations. Here, we show the comprehensive gene mutation profiles of ctDNA in 51 patients with different histological subtypes of stage I–IV ovarian cancer, and their association with clinical outcomes. The ctDNA extracted from pre-treatment patients’ plasma were analyzed using Cancer Personalized Profiling by Deep Sequencing targeting 197 genes. Of 51 patients, 48 (94%) showed one or more non-synonymous somatic mutations, including *TP53* (37.3%), *APC* (17.6%), *KRAS* (15.7%), *EGFR* (13.7%), *MET* (11.8%), *PIK3CA* (11.8%), *NPAP1* (11.8%), and *ALK* (9.8%). The most frequently mutated genes were as follows: *TP53* in high-grade serous carcinoma (66.7%), *APC* in clear cell carcinoma (30.8%), PIK3CA in endometrioid carcinoma (40%), and *KRAS* in mucinous carcinoma (66.7%). Higher cell-free (cf)DNA concentration significantly correlated with worse progression-free survival (PFS) in all patients as well as stage III–IV patients (*p* = 0.01 and 0.005, respectively). Further, patients with any pathogenic mutations showed significantly worse PFS (*p* = 0.048). Blood tumor mutational burden detected from ctDNA did not significantly correlate with the histological subtypes or survival. Collectively, clinico-genomic profiles of individual ovarian cancer patients could be identified using ctDNA and may serve as a useful prognostic indicator. These findings suggest that ctDNA-based gene profiling might help in establishing personalized therapeutic strategies.

## 1. Introduction

Ovarian cancer is often diagnosed at an advanced stage. In addition, it carries the worst prognosis among gynecological malignancies [1,2]. The standard therapy for the disease is primary debulking surgery followed by platinum-based chemotherapy. Generally, treatment options are recommended on the basis of disease stage or histologic subtypes. Although several targeted therapy agents have been applied recently, including the vascular endothelial growth factor inhibitor bevacizumab and the poly-(ADP-ribose) polymerase inhibitor olaparib [3,4], long-term prognosis has not significantly improved [5]. Thus, new personalized therapeutic strategies based on individual molecular or the genetic profile of each patient are needed, which are independent of conventional tumor histological characteristics [6].

Recent advances in cancer genomic medicine have enabled gene-profiling of individual tumors using various multiplex gene panels from tumor tissue-derived DNA samples, and personalized treatment based on the profile data has been applied clinically in some solid cancers. However, surgical tumor biopsy is invasive and sometimes difficult to obtain in advanced/recurrent ovarian cancer patients. Recently, liquid biopsy using circulating tumor DNA (ctDNA), which can analyze repeatedly and in real time, has attracted attention as a non-invasive technique [7,8,9,10,11]. Thus, this technique is an ideal alternative to tumor biopsies, and has been used as a diagnostic, prognostic, and therapeutic decision-making tool, especially in lung cancer (to detect EGFR or KRAS mutations) and colorectal cancer (to detect RAS mutations) [12,13,14]. Furthermore, results from the ctDNA-based genetic analysis have the benefits of not being influenced by intra-tumoral heterogeneity, compared with the tissue-based analysis, and thus could aid the design of effective treatment strategies [15,16]. Consequently, many reports regarding ctDNA analysis have recently been exploding to investigate the feasibility of this technique in establishing a new therapeutic strategy for cancers. However, although extensive research into the genetic profiling of ovarian cancers has been conducted using tumor tissue-derived DNA sequencing [17], the ctDNA liquid biopsy technique has been rarely used thus far in the field of gynecologic oncology.

The first next-generation sequencing (NGS)-based method called Cancer Personalized Profiling by Deep Sequencing (CAPP-Seq) allows for economical and ultrasensitive detection of ctDNA at a low ctDNA input level [18,19]. This technique is clinically applied to patients with different types cancer for detecting somatic mutations in ctDNA [20,21,22].

There have been few reports about the comprehensive gene mutation analysis of ctDNA samples in ovarian cancer, of which most of the analyses were performed only in high-grade serous carcinoma (HGSC) patients [23,24,25]. In our previous study, we showed for the first time the feasibility of ctDNA analysis using CAPP-Seq in gynecological cancers including ovarian cancer, cervical cancer, endometrial cancer, and metastatic colon cancer to the ovary [26,27]. Moreover, we recently demonstrated gene mutation profiles and their monitoring in 10 advanced ovarian cancer patients during neoadjuvant chemotherapy using CAPP-Seq-based liquid biopsy [28]. However, detailed ctDNA-based gene profiling in individual ovarian cancer patients with various disease stage or histological subtypes has not yet been fully elucidated.

Therefore, in the present study, we conducted a comprehensive gene mutation profiling of ctDNA using CAPP-Seq in a larger number of ovarian cancer patients and evaluated its association with clinicopathological and prognostic outcomes to clarify its clinical utility in genetic profile-based personalized therapeutic strategies. Additionally, we examined blood ctDNA-based tumor mutational burden (bTMB), the number of missense mutations per mega base, which might be surrogate for the overall neoantigen load arising as a result of tumor-specific mutations [29,30], and attempted to show the correlation of bTMB with clinical outcomes in ovarian cancer patients.

## 2. Results

### 2.1. Flow Chart of Patients’ Enrollment and Clinical Characteristics

**Figure 1:**
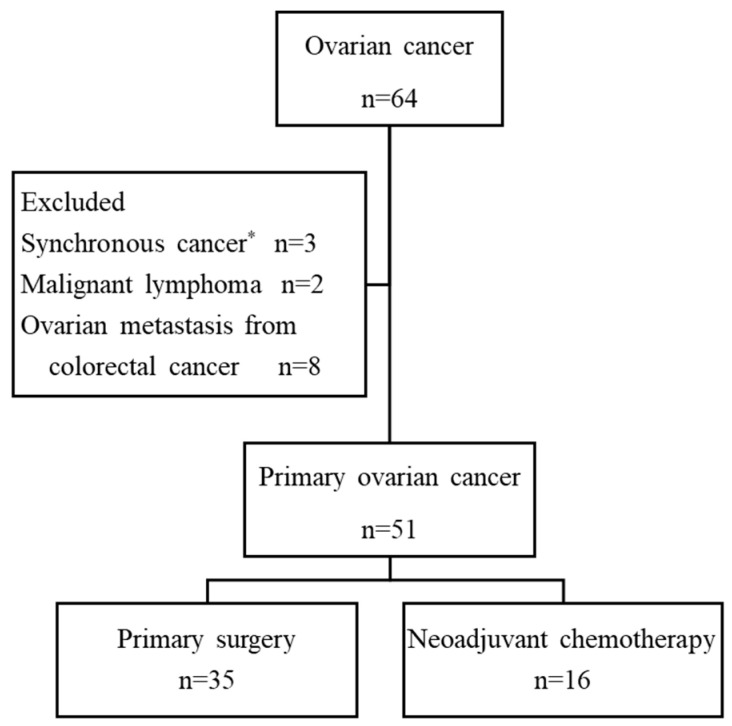
Flowchart of enrolled patients. * Ovarian cancer accompanied with endometrial cancer (n = 1), breast cancer (n = 1), colorectal cancer (n = 1).

A total of 64 patients with ovarian cancer were initially enrolled. Thirteen patients were excluded due to the final pathological diagnosis of synchronous cancer accompanied by other cancers (n = 3), malignant lymphoma (n = 2), or metastatic carcinoma from colorectal cancer (n = 8). The remaining 51 patients were eligible for the study. Of the 51 patients, 35 patients (69%) were treated by primary surgery and 16 (31%) were treated with neoadjuvant chemotherapy (NAC) (Figure 1).

Clinicopathological characteristics in 51 patients are summarized in Table 1. The median age at diagnosis was 60 years (range: 28–82). A total of 15 patients were stage I, 5 were stage II, 23 were stage III, and 8 were stage IV, and 61% were diagnosed at advanced stages III/VI. Precisely 24 of the patients (47%) were diagnosed with high-grade serous carcinoma (HGSC), followed by 13 patients with clear cell carcinoma, 5 with endometrioid carcinoma, and 3 with mucinous carcinoma. Of the 16 patients receiving NAC, 3 patients did not receive post-NAC surgery because of their poor performance status; thus, they were not histologically diagnosed.

### 2.2. Association of cfDNA Levels with Clinical Stage and Histological Subtype

**Figure 2:**
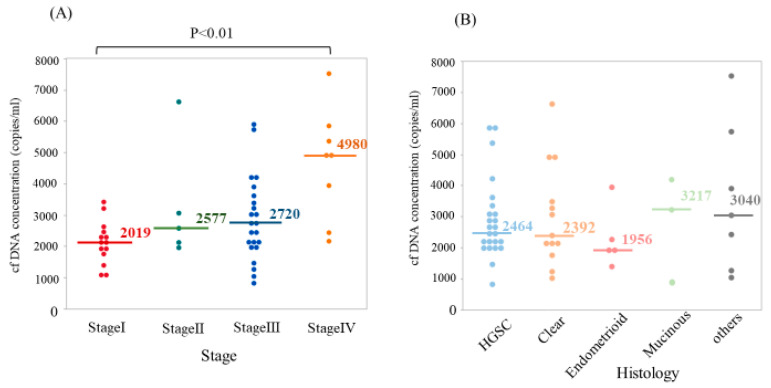
Plasma cell-free DNA (cfDNA) concentration according to disease stage and histological type in ovarian cancer. (**A**) Concentration of circulating tumor DNA (ctDNA) in stage IV was significantly higher compared with stage I (*p* < 0.01). (**B**) There were no significant differences in the level of ctDNA concentration among histological subtypes. *HGSC*: high grade serous carcinoma.

First, we measured cell-free (cf)DNA concentrations in pre-treatment plasma for each patient and compared them among the groups with different disease stage or histological subtype. The cfDNA level of each patient was enough for detection and for further gene mutation analysis, even in stage I disease. The median cfDNA concentration was 2451 copies/mL. As shown in Figure 2A, the concentration of cfDNA in stage IV was significantly higher compared with stage I (*p* < 0.01). In contrast, there were no significant differences in the level of cfDNA concentration among the histological subtypes (Figure 2B).

### 2.3. Profiling of Genetic Somatic Mutation in Ovarian Cancer Using CAPP-Seq

**Figure 3:**
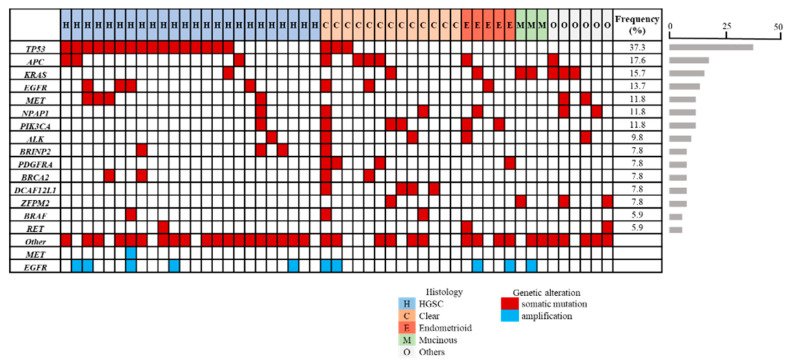
Profiling of genetic mutation in 51 ovarian cancer patients using liquid biopsy-based Cancer Personalized Profiling by Deep Sequencing (CAPP-Seq). Of the 51 patients, 48 (94%) showed one or more non-synonymous somatic mutation. The mutations that occurred in three or more patients are shown. Cases are arranged in the panel from the left to right by histological subtypes. Different mutation types are color-coded. Red represents non-synonymous somatic mutations, and blue represents amplification. The percent of patients with each mutation was indicated by horizontal bars in the left side of the panel.

To identify the individual gene mutation profile for each patient, we analyzed ctDNA obtained from pre-treatment blood samples by CAPP-Seq using gene-sequencing panel containing 197 target genes. Among the 51 patients, 48 (94%) showed one or more non-synonymous somatic mutation. The full details of mutations that occurred in three or more patients are shown in the Figure 3 panel. Frequencies of non-synonymous somatic mutations detected from all patients were *TP53* (37.3%), *APC* (17.6%), *KRAS* (15.7%), *EGFR* (13.7%), *MET* (11.8%), *PIK3CA* (11.8%), *NPAP1* (11.8%), and *ALK* (9.8%). *BRCA1* mutation was detected in two cases (*HGSC* n = 2), and *BRCA2* mutation was detected in four cases (*HGSC* n = 2, clear n = 2). *MET* amplification was detected in one patient with *HGSC*, and EGFR amplification was detected in 10 cases (19.6%) (*HGSC* n = 5, clear n = 2, endometrioid n = 2, mucinous n = 1).

**Figure 4:**
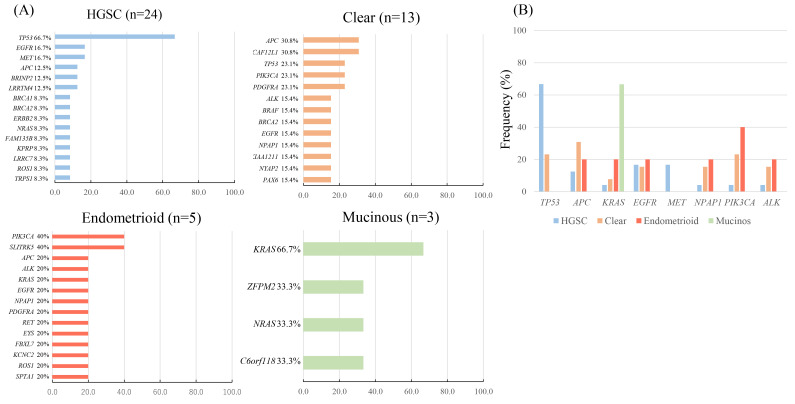
Frequencies of genetic mutation according to histological subtype. Bar graph represented percentage of patients with each mutation. (**A**) Frequencies of genetic mutations in four histological types, high grade serous (*HGSC*), clear, endometrioid, and mucinous carcinoma. (**B**) Frequencies of the top eight genetic mutations (*TP53*, *APC*, *KRAS*, *EGFR*, *MET*, *NPAP1*, *PIK3CA*, and *ALK*) according to histological subtype.

Next, we examined the types of somatic mutation and their frequencies in different histological subtypes (Figure 4). The most frequently mutated gene in *HGSC* was *TP53* (66.7%), which was significantly higher than non-*HGSC* (*p* = 0.004), followed by *EGFR* (16.7%) and *MET* (16.7%). In clear cell carcinoma, *APC* and *DCAF12L1* were mutated preferentially (30.8%), followed by mutations in *TP53*, *PIK3CA*, and *PDGFRA* mutations (23.1%). In endometrioid carcinoma, *PIK3CA* and *SLITRK5* mutations were detected frequently (40%). In mucinous carcinoma, *KRAS* mutation (p.G12D) were detected in two cases (66.7%).

### 2.4. The Concordance of TP53 Mutations between ctDNA and Tissue DNA

To validate the concordance of genomic alternations between ctDNA and tissue DNA, we analyzed tissue DNA of 22 *HGSC* patients, excluding two patients who did not undergo debulking surgery because of poor performance status, using Ion AmpliSeq Cancer Hotspot Panel v2 (CHPv2) (50 genes, 207 amplicons; Life Technologies). We focused on *TP53* mutations that were most frequently detected in *HGSC* patients by CAPP-Seq. *TP53* mutations in tissue DNA were detected in 16/22 patients (72%), and synchronous *TP53* mutations both in tissue DNA and ctDNA were detected in 13/16 patients (81%) (Table S1).

### 2.5. Association of Blood Tumor Mutation Burden (bTMB) with Disease Stage and Histological Subtype

**Figure 5:**
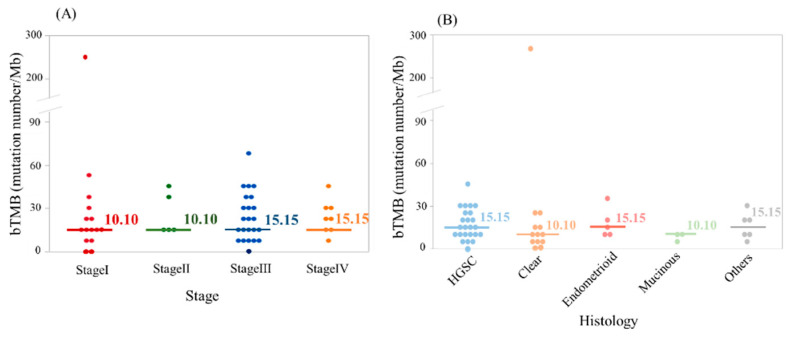
Blood tumor mutational burden (bTMB) according to disease stage and histological type. There were no significant differences in the pre-treatment baseline bTMB levels among groups with different disease stage (**A**) and histological type (**B**).

In this study, bTMB was calculated by the number of non-synonymous mutation number per Mb. The median bTMB was 12.62 (0.00–267.67) mutations/Mb. There were no significant differences in the pre-treatment baseline bTMB level among groups with the different clinical stage or histological subtype (Figure 5A,B).

### 2.6. Impact of cfDNA Concentration and Pathogenic Mutations on Patient Survival

**Figure 6:**
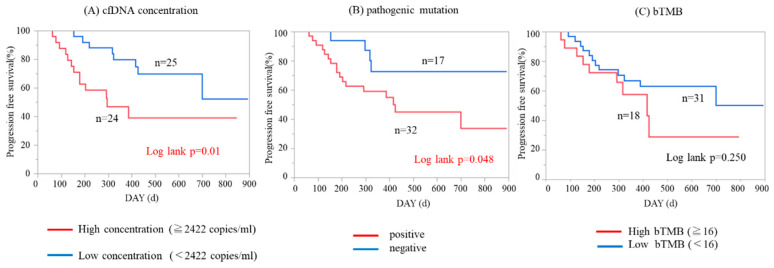
Kaplan–Meier analysis of progression-free survival patients in all ovarian cancer patients (n = 49). (**A**) Patients with higher cfDNA concentration had significantly worse progression-free survival (PFS; *p* = 0.01 by log-rank test). (**B**) Patients with one or more pathogenic mutations had significantly worse PFS (*p* = 0.048 by log-rank test). (**C**) bTMB level was not significantly associated with PFS (*p* = 0.250).

**Figure 7:**
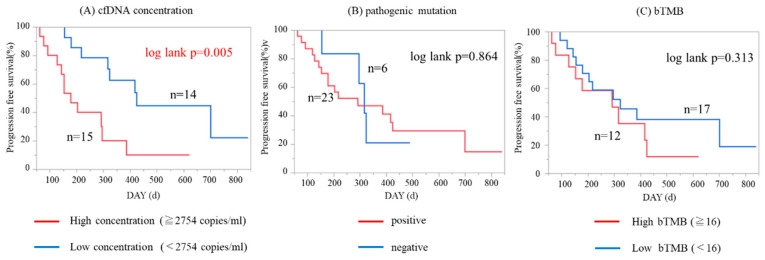
Kaplan–Meier analysis of progression-free survival (PFS) patients in stage III–IV ovarian cancer patients (n = 29). (**A**) Patients with higher cfDNA concentration had significantly worse PFS (*p* = 0.005 by log-rank test). (**B**) Presence/absence of pathogenic mutation did not significantly associate with PFS (*p* = 0.864). (**C**) bTMB level was not significantly associated with PFS (*p* = 0.313).

To determine whether ctDNA-based genetic profile was associated with clinical outcome, we analyzed the impact of cfDNA concentration, presence/absence of pathogenic mutations, and the level of bTMB on survival in 49 patients whose clinical follow-up data could be monitored. We defined whether any of our detected non-synonymous somatic mutations by CAPP-Seq were pathogenic or not, according to the Catalogue of Somatic Mutations in Cancer (COSMIC) database, which is the world’s largest and most comprehensive resource for exploring the impact of somatic mutations in human cancers. With respect to cfDNA concentration and bTMB, we divided patients into two groups using the median values. As shown in Figure 6A,B, patients with higher cfDNA concentrations and patients with any one or more pathogenic mutations showed significantly worse progression-free survival (PFS; *p* = 0.01 and 0.048 by log-rank test, respectively). In contrast, bTMB level was not significantly associated with PFS (Figure 6C). Next, we performed the analysis focusing on 29 patients with stage III/IV disease. Higher cfDNA concentration was significantly associated with worse PFS in advanced-staged patients (Figure 7A; *p* = 0.005 by log-rank test). The presence/absence of pathogenic mutations or bTMB levels did not significantly associate with PFS in stage III/IV patients (Figure 7B,C).

### 2.7. Monitoring of Changes in ctDNA-Based Genetic Profiles During Treatment Course

**Figure 8:**
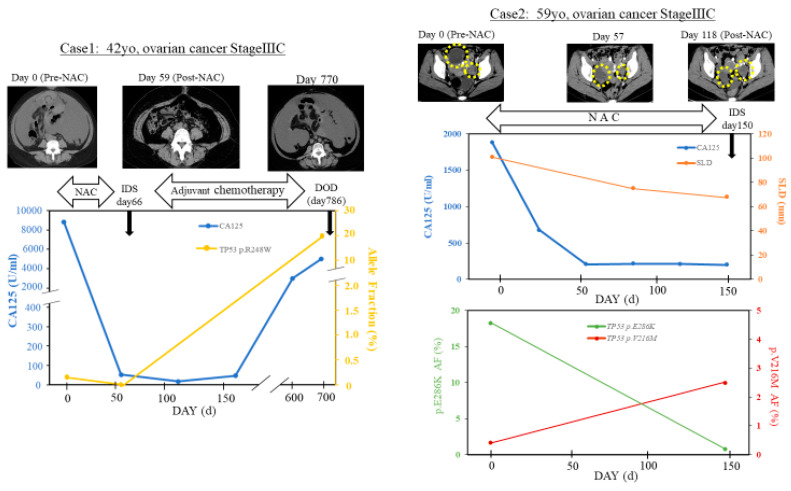
Monitoring ctDNA in neoadjuvant chemotherapy (NAC)-treated ovarian cancer patients. The figures showed the time course of allele frequency of ctDNA, serum CA125 level, and sum of the longest diameters (SLD; only in case 2) with their clinical events and treatments as well as changes in computed tomography (CT) images. In case 1, *TP53* R248W level in ctDNA (yellow line) was not detected after NAC alongside response to treatment. The allele fraction of *TP53* R248W in ctDNA increased during the serum CA125 level (blue line) along with progressive disease. In case 2, two site mutations of *TP53* (p.E286K, V216M) were detected before NAC. The patient responded initially to NAC, but SLD of the tumor (orange) and the serum CA125 level (blue) remained stable. After five cycles of NAC, the allele fraction of *TP53* p.E286K (green line) decreased, while the allele fraction of *TP53* p.V216M (red line) increased. SLD: sum of the longest diameters.

We examined the utility of ctDNA-based liquid biopsy for real-time treatment monitoring; two representative cases whose samples were collected repeatedly are shown in Figure 8.

Case 1 was diagnosed with stage IIIC ovarian cancer and treated with three cycles of NAC (carboplatin plus docetaxel). The patient responsiveness was great with NAC, as shown in the CT images. The *TP53* p.R248W mutation in ctDNA was not detectable after NAC (day 66), while serum CA125 level remained positive on day 66 and later became negative by day 144. She received interval debulking surgery (IDS), followed further by adjuvant chemotherapy; however, serum CA125 level increased along with progressive disease, and the individual died approximately 2 years after IDS. The allele fraction of *TP53* R248W in ctDNA increased during disease progression.

Case 2 was diagnosed with stage IIIC ovarian cancer and treated with NAC (carboplatin plus paclitaxel). Two site *TP53* mutations (p.E286K, V216M) were detected before NAC. The patient initially responded to NAC as shown in computed tomography (CT) images along with a decrease in the sum of the longest diameters (SLD) of the tumor and the serum CA125 level. After three cycles of NAC, the patient showed progressive disease due to new peritoneal metastasis, although serum CA125 level remained stable around 200 U/mL. After five cycles of NAC, the allele fraction of *TP53* p.E286K decreased, while the allele fraction of *TP53* p.V216M increased.

## 3. Discussion

In the present study, we utilized CAPP-Seq, an ultrasensitive NGS-based liquid biopsy approach, for blood-derived ctDNA samples obtained from patients with ovarian cancer of different stages and major histopathological subtypes. We characterized comprehensive genomic profiles including cfDNA concentration, genetic alteration, and bTMB for individual cases and subgroups in the ctDNA samples from 51 ovarian cancer patients. We also demonstrated that higher levels of cfDNA concentration and detection of more than one non-synonymous pathogenic mutations associated with worse PFS in patients with ovarian cancers. These findings suggest that ctDNA liquid biopsy might be useful not only as a non-invasive tumor genotyping tool but also as a predictive biomarker of prognosis in clinical situation for ovarian cancer.

Consistent with the previous reports showing that cfDNA concentrations corresponded to the tumor burden and cancer stage [31,32], we found that the cfDNA concentration increased with advancement of ovarian cancer stage, while significant difference was seen in only stage I vs. stage IV without significant difference among histological subtypes. In our study, we demonstrated that higher cfDNA concentration was significantly associated with worse PFS, both in the whole cohort and in the advanced stage (III/IV) group. Indeed, although cfDNA concentration may consist of both non-tumor- and tumor-derived cfDNA, our results suggest that cfDNA concentration might be able to predict prognosis in ovarian cancer patients, as previously reported in many solid cancers [33,34,35,36].

In addition to analyzing cfDNA concentration, we analyzed comprehensive gene alterations from ctDNA using the ultrasensitive NGS method CAPP-Seq, targeted hybrid captures with high-throughput sequencing, and a specialized bioinformatics workflow technique. To the best of our knowledge, the present study is the first to demonstrate ctDNA-utilized genomic profiling, not only in HGSC but also in other major histological subtypes of ovarian cancer. There have been only a few studies about a comprehensive gene mutation analysis of ctDNA in the gynecological oncology field, and they involved its usage in a few patients [23,24,25]. Phallen et al. [23] used targeted error correction sequencing, which allowed direct ultrasensitive evaluations of 58 cancer-related genetic alterations in order to analyze ctDNA from four types of cancers (colorectal, lung, ovarian, and breast cancer). In their study, out of the 42 HGSC patients, 27 (64%) had at least one detectable somatic mutation in their plasma ctDNA (range: 1–3 mutations/sample), and approximately 50% of the ovarian cancer patients had *TP53* mutations. Cohen et al. [24] used CancerSEEK, a combined assay for genetic alterations in 16 cancer-related genes and 41 protein biomarkers, in order to analyze eight types of cancers (colorectal, lung, ovarian, breast, liver, stomach, pancreatic, and esophageal cancer). Although the 54 HGSC patients included in the latter study had detectable somatic mutations in their plasma ctDNA, only one somatic mutation was detected in each patient, and 46 (85%) patients had *TP53* mutations. Arend et al. [25] used a basic NGS method (the Ion AmpliSeq cancer hotspot panel v2) for ultra-deep targeted sequencing of ctDNA from 14 patients with advanced HGSC patients (stage III/IV). Although they detected 12 variants in matched tumor and plasma ctDNA samples, it was considered likely that all 12 of these variants were germline mutations. In our study, most of the ovarian cancer patients (48/51; 94.1%) had at least one detectable non-synonymous somatic mutation in their ctDNA, and we could detect the gene mutation patterns according to independent histological subtypes.

Our analysis showed that the *TP53* mutation detected in the ctDNA was the most frequent in whole study cohort (19/51; 37.3%), and in most of the HGSC cases (16/24; 66.7%), which is less than previous tissue DNA studies [25]. However, the detection rate of *TP53* mutation in ctDNA from HGSC patients was reported at about 40–60% in previous ctDNA studies [25,28]. To validate the concordance of genomic alternations between tissue DNA and ctDNA, we analyzed tissue-DNA of 22/24 HGSC patients by focusing on *TP53* mutations. *TP53* mutation in tissue DNA was detected in 16/22 patients (72%), which was consistent with the data reported by previous studies in Japanese ovarian cancer patients [37,38]. Furthermore, our data showed that synchronous *TP53* mutations both in tissue DNA and ctDNA were detected in 13/16 patients (81%). These results suggest that mutational analysis of ctDNA in our study might mostly reflect tumor mutational landscape.

Besides HGSC, *TP53* mutation was observed in ovarian clear cell carcinoma (OCCC) cases (3/13; 23.1%), with no detection in other histological subtypes. *PIK3CA* mutation in ctDNA was frequently detected in endometrioid carcinoma cases (2/5; 40.0%), followed by OCCC cases (3/13; 23.1%), which is consistent with previous tissue DNA studies [39,40]. Previous reports suggested that *PIK3CA* mutations might predict sensitivity to treatment with PI3K/AKT/mTOR inhibitors, such as everolimus, in multiple tumor types including gynecologic cancer [41,42]. In our study, all six *PIC3CA*-mutated cases harvested pathogenic mutation (codons 542/545/1047), a hotspot mutation with known transforming capacity with sensitivity to mTOR inhibitor everolimus, which might be the therapeutic target for personalized medicine. *APC* mutation in ctDNA was detected in OCCC cases (4/13; 30.8%), followed by endometrioid cases (1/5; 20.0%) and HGSC cases (4/24; 12.5%), although there was only one case with the known pathogenic *APC* mutation. The result of *APC* mutation is controversial, compared with previous tissue DNA studies [17,43]; thus further study is needed to clarify the role of *APC* mutation in ovarian cancer. Our study detected the *KRAS* gene mutation in ctDNA out of eight cases in the total study cohort (8/51; 15.7%), and most of the cases harbored pathogenic mutations (codons 12/13/61) (7/8; 87.5%). *KRAS* mutations have been reported to be poor prognostic markers in colorectal, lung, and pancreatic cancer [12,44,45]. Consequently, in our study, four *KRAS*-mutated cases (codon 12) died in 3–4 months after diagnosed of ovarian cancer. Thus, *KRAS* mutation in codon 12 might be a worse prognostic ctDNA biomarker. In addition, *BRCA1* mutation was detected in two cases, and *BRCA2* mutation was detected in four cases, although these *BRCA* mutations were not considered to be pathogenic on the basis of the COSMIC database. Our study showed that *EGFR* gene amplification in ctDNA was detected in 10 patients (19.6%). In previous reports, *EGFR* gene amplification was associated with poor patient outcome in ovarian cancer and was detected in 4–22% of cases [46]. Hence, *EGFR* gene amplification in ctDNA might be a predictive marker for patients’ responsiveness to treatment in ovarian cancer.

Interestingly, we demonstrated the association between the pathogenic somatic mutations and clinical outcomes, which have been previously reported in other solid cancer [36,47,48]. In our study, a patient having one or more pathogenic somatic mutations, defined according to the COSMIC database, showed significantly poor PFS, as compared with patients without the pathogenic mutations. This suggested the pathogenic somatic mutation may play an important role as disease progression of ovarian cancer.

Indeed, our results demonstrated the possibility of performing ctDNA liquid biopsy for monitoring treatment responses, including "tumor evolution" [49]; that is, evolutionary changes in tumor heterogeneity or clonality in NAC-treated patients with advanced ovarian cancer (Figure 8). In ctDNA analysis of our case 1, we detected a reduction in the *TP53* (R248W) mutation level after NAC treatment, and an increase in the same *TP53* (R248W) mutation level after disease progression. In ctDNA analysis of our case 2, we detected a reduction in the *TP53* (E286K) mutation level after NAC treatment, while another *TP53* (V216M) mutation level was increased, which might enhance the populational change of *TP53*-mutated cancer cells. This observation suggested that these mutations might have originated from different sub-clones and the clonal revolution might occur under the selective pressure of therapeutic intervention that finally leads to the expansion of resistant clones. Thus, CAPP-Seq, using ctDNA samples, might be a useful tool for non-invasive tumor genotyping, as well as therapeutic response monitoring and the detection of evolutionary changes in tumor heterogeneity.

Gandara et al. reported that high bTMB (≧16 Mb) was clinically responsive to anti-PD-L1 therapy in non-small cell lung cancer [50]. This study suggested the association of bTMB and clinical outcomes in ovarian cancer for the first time. We reported previously that bTMB might be a new biomarker for monitoring treatment during NAC in advanced ovarian cancers [28], but the mechanism of the association of bTMB with efficacy of chemotherapy nor immunotherapy remains unclear in ovarian cancer. In our current analysis of bTMB among all the clinical stages and different histological subtypes, although there were no significant differences in the pre-treatment baseline bTMB in each group, or bTMB association with PFS, we could detect some populations of highly mutated tumors in each group (Figure 5), which might be the therapeutic target for anti-PD-L1/PD-1 therapies [50]. Further experiments are needed to evaluate whether bTMB might serve as a non-invasive alternative biomarker for clinical benefit to ovarian cancer patients, especially in terms of advanced stage.

Despite the success of our findings, our study does not preclude limitations. First, this was a retrospective study with a small sample size, especially for such relatively rare tumor subtypes (e.g., clear, endometrioid, and mucinous); hence, further studies with a larger patient population are needed to determine the predictive biomarker from the ctDNA gene profiling. Second, we did not compare our ctDNA data with the results from the tumor DNA. Our previous feasibility study showed that, in three of four ovarian cancer samples, the same pathogenic mutations were detected in both the plasma ctDNA and tumor DNA [27]. Furthermore, in the present study, we demonstrated the high concordance rate (81%) of *TP53* mutations between ctDNA and tissue DNA in HGSC patients. Although CAPP-Seq has not been applied to a tumor tissue sample thus far, this technique needs to be expanded not only to the ctDNA analysis but also to the tumor DNA analysis. Thus, we require further experiments using tumor DNA to validate accurate detection of somatic mutations in ctDNA. Finally, it is technically arduous to strictly differentiate ctDNA in the presence of cfDNA from normal tissues and blood cells. As previously reported, CAPP-Seq enabled the detection of tumor-specific mutations in ctDNA for a broad range of cancers [18,19,26,27,28].

In conclusion, we demonstrated that comprehensive gene mutation profiling of ctDNA could be performed successfully by CAPP-Seq in ovarian cancer patients, as well as the fact that the determination of cfDNA levels might be a new prognostic biomarker in ovarian cancer patients. If blood samples were collected at a greater number of time points, changes in the allele frequency can also be detected in detail and liquid biopsy might enable early detection of tumor relapse/progression and assessment of post-treatment minimal residual disease (MRD). Although genetic profiling using tumor DNA in gynecological cancers was reported [17], thus far, obtaining sufficient samples with tumor biopsy is often difficult, particularly in cases of advanced/recurrent ovarian cancer. Therefore, comprehensive genetic profiling of noninvasive ctDNA-based liquid biopsy, which might reflect heterogeneity in real time, might provide effective treatment for individual patients and monitoring response to treatment in the field of ovarian cancer.

## 4. Materials and Methods

### 4.1. Patients and Samples

We conducted a retrospective study enrolling 51 female patients (median age = 60 years) who had been newly diagnosed with ovarian cancer and had undergone primary surgery or neoadjuvant chemotherapy (NAC) between May 2017 and March 2020 at Wakayama Medical University Hospital, Japan. The patients’ primary cancers were staged according to the International Federation of Gynecology and Obstetrics (FIGO) surgical staging criteria. The postoperative pathological diagnosis was designated according to the criteria of the World Health Organization (WHO) classification. Patients who underwent NAC were initially diagnosed and staged using the clinical findings of images acquired from computed tomography (CT) scan, magnetic resonance imaging (MRI), and positron emission tomography/computed tomography (PET/CT), along with the results from cytology of ascitic or pleural fluids. Most patients receiving NAC were histologically diagnosed after the post-NAC debulking surgery. The majority of the patients, except the stage IA cases, received 3−6 cycles of paclitaxel (175 mg/m^2^, day 1) and carboplatin (5 areas under the curve, day 1) with or without bevacizumab (15 mg/kg, day 1) every 21 days as postoperative therapy or NAC. In cases showing resistance to the first-line chemotherapy, second-line regimens, which included mainly cisplatin (60 mg/m^2^, day 1) and irinotecan (60 mg/m^2^, day 1/8/15), were administered.

Blood samples were obtained before the primary surgery. In NAC cases, blood samples were obtained pre-, during, and post-NAC.

This study was conducted with the approval of the ethics committee of Wakayama Medical University (authorization number: 2025) and Kindai University Faculty of Medicine (authorization number: 29-066). Written informed consent was obtained from each of the patients. The study was carried out in compliance with the principles of the Helsinki Declaration update of 2008.

### 4.2. Circulating Tumor DNA Extraction

Peripheral blood samples (8.5 mL) from the patients were collected in cell-free DNA collection tubes (Roche Diagnostics, Indianapolis, IN, USA). DNA was extracted from 4 mL of plasma using an AVENIO cfDNA isolation kit (Roche Diagnostics) according to the manufacturer’s instructions, and cfDNA was eluted in 65 μL elusion buffer. The quality and quantity of cfDNA were verified using the PicoGreen dsDNA assay kit (Life Technologies, Carlsbad, CA, USA). The extracted cfDNA was stored at −80 °C until the analysis.

### 4.3. Circulating Tumor DNA Sequencing

The CAPP-Seq of ctDNA (10−50 ng) was performed using the AVENIO ctDNA surveillance kit targeting 197 genes (Roche Diagnostics) as previously described [26,27,28]. The purified libraries were pooled and sequenced on Illumina NextSeq 500 (Illumina, San Diego, CA, USA) using 300-cycle high output kit. Variants were called with the AVENIO ctDNA Analysis Software (Roche Diagnostics), which includes bioinformatics methods from CAPP-Seq [18] and integrated digital error suppression [19]. Genetic variants previously cataloged by the Exome Aggregation Consortium at a frequency of ≥1% were excluded, and only non-synonymous single nucleotide variants (SNVs), insertions–deletions (Indels), copy number variations (CNVs), and gene fusions involving 197 cancer-related genes were extracted. The blood tumor mutational burden (bTMB) in each sample was evaluated as the number of non-synonymous mutations number per Mb.

### 4.4. Tumor DNA Extraction

Formalin‑fixed paraffin‑embedded (FFPE) specimens were subjected to nucleic and extraction. DNA was purified with the use of an AllPrep DNA/RNA FFPE kit (Qiagen, Inc., Valencia, CA, USA), according to the manufacturer’s instructions. The quality and quantity of the DNA were verified with the use of a NanoDrop 2000 device (Thermo Scientific, Wilmington, DE, USA) and PicoGreen dsDNA assay kit (Thermo Fisher Scientific, Inc. Waltham, MA, USA). The extracted DNA was stored at −80 °C until the analysis.

### 4.5. Tumor DNA Sequencing

NGS libraries were prepared according to the manufacturer’s protocol with the Ion AmpliSeq Library kit version 2.0 (Thermo Fisher Scientific). For DNA sequencing, 10 ng of DNA was subjected to multiplex PCR amplification with the use of an Ion AmpliSeq Cancer Hotspot Panel (CHPv2) primer pool (50 genes, 207 amplicons; Thermo Fisher Scientific). After multiplex PCR, Ion Xpress Barcode Adapters (Thermo Fisher Scientific) were ligated to the PCR products, which were then purified with the use of Agencourt AMPure XP beads (Beckman Coulter, Brea, CA, USA). The purified libraries were pooled and then sequenced with the use of an Ion Torrent S5 instrument and Ion 550 Chip Kit (all from Thermo Fisher Scientific). DNA sequencing data were accessed through the Torrent Suite v.5.12 program (Thermo Fisher Scientific). Reads were aligned against the hg19 human reference genome, and variants were called with the use of Variant Call Format ver. 5.12. Raw variant calls were filtered with depth of coverage of <19 and were manually checked using the integrative genomics viewer (IGV; Broad Institute, Cambridge, MA, USA). Germline mutations were excluded with the use of the Human Genetic Variation Database (http://www.genome.med.kyoto-u.ac.jp/SnpDB) [51].

### 4.6. Statistical Analysis

Statistical analyses were performed using JMP Pro statistical software version 13.1.1 for Windows (SAS Institute Inc, Cary, NC, USA). Statistical comparisons between the groups were performed using the Mann−Whitney *U* test or the Kruskal−Wallis test. Associations of the gene alternations with histology were evaluated using Fisher’s exact test. Progression-free survival (PFS) was defined as the time from the date of diagnosis of ovarian cancer to the date of disease progression and was calculated using the Kaplan–Meier method. Between-group comparisons in survival analysis were performed using the log-rank test. Differences were considered to be significant when *p*-values < 0.05.

## 5. Conclusions

Our study demonstrated clinico-genomic profiles of individual ovarian cancer patients could be identified using ctDNA and may serve as a useful prognostic indicator. ctDNA-based gene profiling might help in establishing personalized therapeutic strategies.

## Figures and Tables

**Table 1:** Clinicopathological characteristics in of 51 ovarian cancer patients.

Age (Median)	60 (28–82)
Stage	No. (%)
I	15 (29)
II	5 (10)
III	23 (45)
IV	8 (16)
Histology	No. (%)
High-grade serous carcinoma (HGSC)	24 (47)
Clear cell carcinoma	13 (25)
Endometrioid carcinoma	5 (10)
Mucinous carcinoma	3 (6)
Others*	6 (12)
Treatment	No. (%)
Primary surgery	35 (69)
Neoadjuvant chemotherapy	16 (31)

* Others*: Low grade serous carcinoma n = 1, Squamous cell carcinoma n = 1, Hepatoid carcinoma n = 1, not diagnosed n = 3.

## Supplementary Materials

The following are available online at https://www.mdpi.com/2072-6694/12/11/3382/s1: Table S1: List of *TP53* mutations detected in ctDNA and tissue DNA.
