# Supplementary material for: Comprehensive Gene Mutation Profiling of Circulating Tumor DNA in Ovarian Cancer: Its Pathological and Prognostic Impact
Source: Cancers (Basel). 2020 Nov 16;12(11):3382. doi: 10.3390/cancers12113382 (PMC7697720; doi:10.3390/cancers12113382)
Supplement: Supplementary file 1 [file cancers-12-03382-s001.pdf]

cancers

Supplementary Materials

Comprehensive Gene Mutation Profiling of Circulating Tumor DNA in Ovarian Cancer: Its Pathological and Prognostic Impact

Tomoko Noguchi, Naoyuki Iwahashi, Kazuko Sakai, Kaho Matsuda, Hitomi Matsukawa, Saori Toujima, Kazuto Nishio, Kazuhiko Ino

Table S1. List of *TP53* mutations detected in ctDNA and tissue-DNA.

patient No.	ctDNA	tissue-DNA
HGSC-01	p.R248W	p.R248W
HGSC-02	not detected	not detected
HGSC-03	p.V216E	p.V216E
HGSC-04	p.R213*	p.R213*
HGSC-05	not detected	not detected
HGSC-06	p.E286K	p,E286K
HGSC-07	p.R175H	p.R175H
HGSC-08	p.Y205C	p.Y205C
HGSC-09	p.R196*	p.R196*
HGSC-10	not detected	not detected
HGSC-11	p.Q136E	p.Q136E
HGSC-12	p.H179Y	p.H179Y
HGSC-13	not detected	not detected
HGSC-14	p.R248W	p.R248W
HGSC-15	c.673-2A>G	c.673-2A>G
HGSC-16	not detected	p.K132R
HGSC-17	not detected	p.E349*
HGSC-18	p.C238Y	p.C238Y
HGSC-19	not detected	not detected
HGSC-20	not detected	p.E171G
HGSC-21	p.R249W	p.R249W
HGSC-22	p.R248W	not detected

The marker indicated the synchronous *TP53* mutations both in tissue-DNA and ctDNA.
